# Overcoming limitations for antibody-based therapies targeting γδ T (Vg9Vd2) cells

**DOI:** 10.3389/fimmu.2024.1432015

**Published:** 2024-07-31

**Authors:** Lucía Paniagua-Herranz, Cristina Díaz-Tejeiro, Adrián Sanvicente, Jorge Bartolomé, Cristina Nieto-Jiménez, Alberto Ocana

**Affiliations:** ^1^ Experimental Therapeutics Unit, Oncology Department, Hospital Clínico San Carlos (HCSC) Instituto de Investigación Sanitaria San Carlos (IdISSC), Madrid, Spain; ^2^ Centro de Investigación Biomédica en Red en Oncología (CIBERONC), Madrid, Spain

**Keywords:** γδT cells, BTN3A, T cell engager, immunotherapy, immunologic response

## Abstract

Therapeutic strategies targeting non-adaptive immune cells are currently in clinical development. γδT cells are a small subtype of T cells (1-10% of total T cells) that mediate their effector function without the necessity of the antigen presenting machinery, and also share functional properties with innate cells. Among the different γδT subtypes, antibodies against Vγ9Vδ2T have reported signs of clinical efficacy in early clinical studies. In this review we describe the biology of this subtype of non-conventional T cells and provide insights into the mechanism of action of novel antibodies that activate these cells. We will focus on antibodies targeting the BTN3A ligand and bi-specific γδT cell engagers. We will review in detail the advantages of these strategies including the potential for overcoming mechanisms of resistance to check point inhibitors, or the much more adequate safety profile compared with agents activating classical T cells. Limitations identified during the first studies in humans and strategies to overcome them will be revised and discussed. Finally, clinical options for future clinical development will be suggested.

## 1 Introduction

Most efforts in cancer research have been focused on the identification of druggable vulnerabilities in cancer cells, with the aim to develop therapeutic strategies against them (1). This has been the case for the identification of genomic alterations such as mutations or copy number variations that induced a survival gain (2, 3). Therapies against those vulnerabilities, including chemical entities acting on protein activating domains, or antibodies targeting extracellular proteins have demonstrated clinical activity (3–5).

Much more recently, boosting the host immune response as a therapeutic strategy, have showed clinical activity with the development of agents targeting immune suppressive molecules like PDL1 or CTLA4 (6). When exploiting the immune system for therapeutic purposes it should be taken in consideration that we are acting on the own host immune response against the tumor and not against transformed cells (7). In this context, two different aspects should be considered: first, the interaction that the tumor cell induces in the immune system and secondly, the immunologic response of each individual patient to the tumor (8).

A great endeavor has been made in the oncology community to identify modulators of the immune response that could be therapeutically exploited to induce an effector immune action. The first attempts focused on boosting the adaptive immunity by stimulating an effector T cell response (9). This was obtained with checkpoint inhibitors (CPI) that released the action of effector T cells. The confirmation that an efficient effector T cell response was able to induce biological activity that translated into clinical efficacy, led to the development of direct strategies to activate T cells (9). Different bi-specific antibodies have been developed acting co-simultaneously on stimulating receptors and immune CPI (10, 11). A step further was the design of T cell engagers that aimed to activate T cells through the CD3 receptor component, bringing in proximity a tumoral cell by binding to a tumor associated antigen (TAA) (12).

However, cells for the innate immune system could also be exploited for therapeutic purposes (13). These cells could be targeted to either induce a direct activation, acting on stimulatory or inhibitory receptors, or through mechanisms involved in the production of an immune suppressive environment (13).

**Figure 1:**
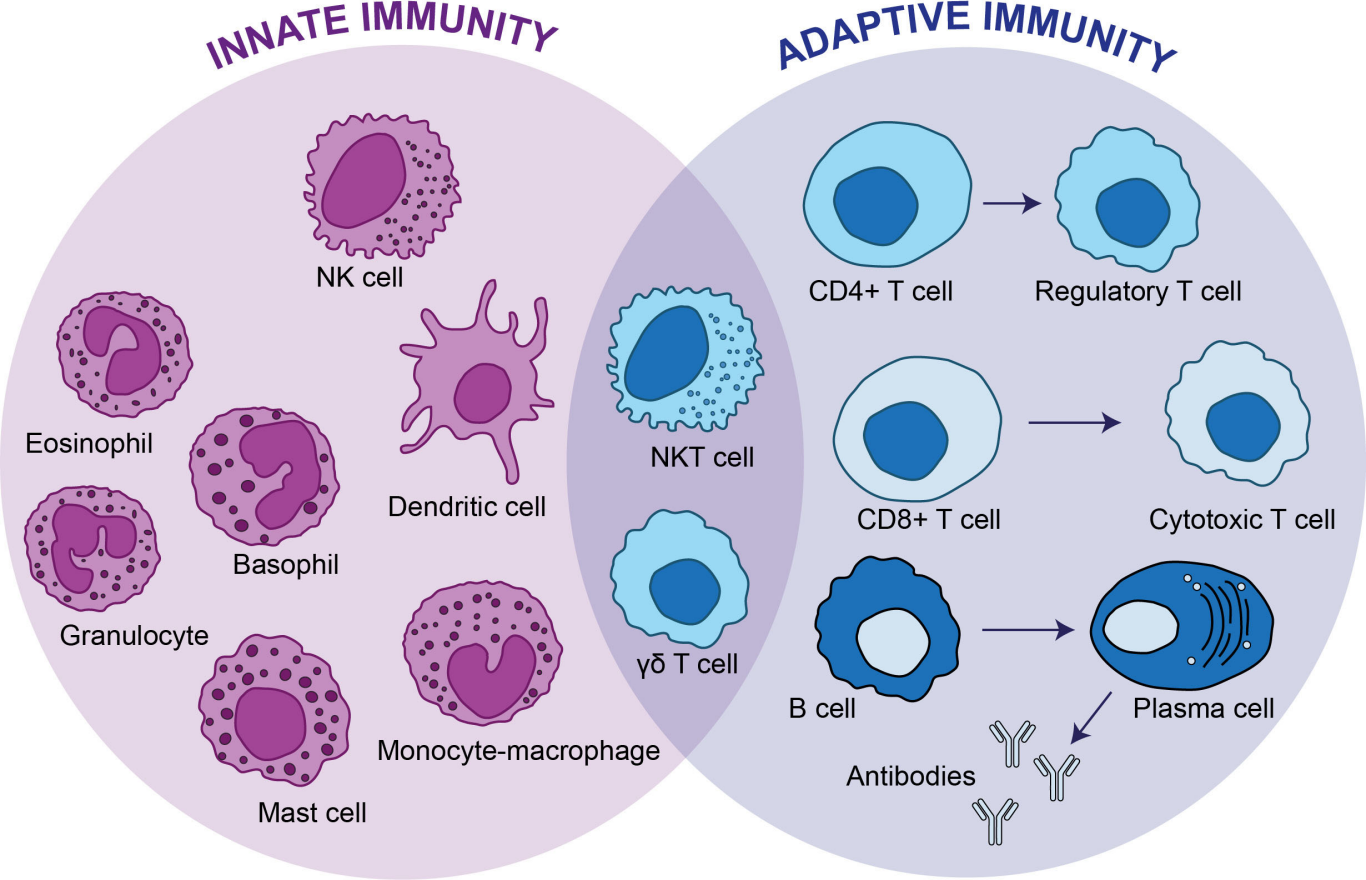
Innate immunity vs adaptive immunity. The innate immunity is the first line of defense and provides a non-specific response against pathogens, whereas adaptive immunity targets specific antigens. The innate immunity consists of dendritic cells, mast cells, eosinophils, basophils, granulocytes and monocytes-macrophages, whereas CD4+ T cells, CD8+ T cells and B cells conforms the adaptive immunity. There are two types of cells, natural killer T cells and γδ T cells that have functions of cytotoxic lymphocytes and overlap between the innate and adaptive immunity.

A particular type of cells that share characteristics between innate and adaptive cells are termed γδ T cells (**Figure 1**). In this article we will review therapeutic strategies to boost the effector function of γδ T cells.

### 1.1 γδ T cells

γδT cells represent a small population (1%-10%) of CD3 T cells (14, 15). This population share features with classical T cells, like the T-cell receptor (TCR), and innate-like properties with natural killer (NK) cells, mucosal-associated invariant T cells, and natural killer T (NKT) cells (15). Although γδ T cells harbor a TCR they are activated in a non–major histocompatibility complex (MHC)–restricted manner. On the other hand, the activation is based on receptor-ligand interactions with a rapid response like innate cells (16, 17).

The previous characteristics just described are the reason why these cells have been called “non-conventional” T-lymphocytes (15). γδ T cells are characterized by harboring γδ chains in the TCR (γδTCR) instead of αβ, like the major population of αβT cells (18). They can be classified in five different types based on how the TCR chains are grouped together, as there are five different δ chains Vδ1, 2, 3, and 5; and seven different γ chains Vγ2, 3, 4, 5, 8, 9, and 11 (19). These subtypes are described in **Table 1**.

**Table 1:** Subsets of human γδ T cells.

Subset	Paired TCR chains	Cellular localization
**Vδ1**	Vγ2, Vγ3, Vγ4, Vγ5, Vγ8 and Vγ9	Skin, intestine, liver, spleen and mucosal tissues
**Vδ2**	Vγ9	Peripheral blood
**Vδ3**	Vγ2, Vγ3	Liver and peripheral blood
**Vδ5**	Vγ4	Peripheral blood

Vγ9Vδ2T represent 95% of all the γδ T cells in peripheral blood and have a clear effector function, therefore their expression in tumors has been associated with favorable outcome (20). In addition, they can be activated through the interaction of the TCR with butyrophilin (BTNA) ligands, inducing a strong immune effector cell response (21, 22). By contrast, the Vδ1T subtype is form of combinations with Vγ9,2,3,4,5,8 (**Table 1**). This subtype is mainly located in the skin and epithelial tissue. Vγ9Vδ2T has a clear antitumoral role while for Vδ1T pro-tumoral function have been described (23). Characteristics of other subtypes are described in **Table 1**.

### 1.2 Effector functions of Vγ9Vδ2T through innate and adaptive mechanisms

As discussed before, Vγ9Vδ2T share mechanisms related to innate and adaptive immunity (13). Vγ9Vδ2T can be activated through the TCR binding with butyrophilin (BTNA) ligand (21, 24). These family of ligands, that also include several isoforms like BTN3A1, BTN3A2, and BTN3A3, are expressed on the surface of modified cells like tumoral or infected cells (22, 25). Of note, both BTN3A2 and BTN3A3 are also expressed in normal tissue (26). Indeed, it has been detected a strong expression of both isoforms in colon, lung and small intestine tissues. However, BTN3A2 expression is higher in malignant cells of several tumor types such as lung or colon among others, compared with normal tissue. BTN3A isoforms seem to be expressed at higher levels in tumor-infiltrating immune cell than in the related cells in peripheral blood (27). Finally, to add more complexity to the biological scenario, some authors have demonstrated that BTN2A1 and BTN3A1 are also expressed on Vg9Vd2 T cells, and can be stimulated by the same means as in other cells (28).

Small pyrophosphate molecules (IPP) that are produced in these tumoral cells, and that are intermediates of the cholesterol synthesis pathway, can activate the BTN3A1 and its intracellular B30.2 signaling domain leading to a conformational change that interact with the Vγ9Vδ2T TCR inducing an effector function (29–32). These IPP molecules have been called phosphoantigens (PAg). Nevertheless, it seems that elevated levels of PAg in tumor cells are not sufficient to produce the full activation of BTN3A1, therefore strategies to increase PAg would be necessary.

Vγ9Vδ2T TCR engagement has recently been shown to be initiated by its interaction with BTN2A1, another member of the BTN family, which directly binds to the Vγ9 chain of the Vγ9Vδ2T TCR through its immunoglobulin V (IgV) domain and forms complexes with BTN3A1 (25, 33). As mentioned, the cytoplasmic regions of BTN3A1 and BTN3A3 contain a pAg-binding B30.2 domain, which is absent from BTN3A2 (34).

The effector T cell function is performed via perforin/granzyme B and Fas ligand cytotoxic pathway (35). Interestingly, this mechanism of activation can be exploited therapeutically through the development of antibodies that by binding to BTN3A induce a conformational change to its activating form.

In addition to this mechanism, Vγ9Vδ2T harbors receptors from innate cells like Natural killer (NK) cells. Among these receptors we can highlight the activating receptor NKGD2 that is triggered through at least one of the eight NKG2D ligands (MHC class I-related chain A/B [MICA/B], that are expressed on transformed cells (23, 36). Other NK receptors, such as NKp30, NKp44, and DNAM-1 (CD226), can also be expressed at varying levels on γδ T cells and contribute to tumor cell recognition and killing (14).

### 1.3 Clinical opportunities targeting Vγ9Vδ2T

Targeting Vγ9Vδ2T provides different opportunities for clinical development. The fact that activation of Vγ9Vδ2T does not depend on the antigen presenting machinery permits the activation of an effector immune response in tumor conditions that lack this mechanism. For instance, PD (L)1 resistant tumors frequently lose HLA-class-I-mediated antigen presentation due to silencing of HLA class I genes, inactivating mutations in β2-microglobulin (encoded by B2M) or other defects in the antigen processing machinery, which can render these tumors resistant to CD8+ T-cell-mediated immunity (37, 38). In addition, Vγ9Vδ2T can be active in tumors that do not have a high grade of genomic instability, high tumor mutational burden or high release of neoantigens, contrary to anti PD (L)1 therapies or those therapies that directly activate conventional T cells (15). Therefore, therapies boosting Vγ9Vδ2T cell response could be evaluated in a wide range of tumors and not only on those considered as immune reactive. In line with this, targeting Vγ9Vδ2T can overcome some of the limitations of anti-PD1 therapies and could overcome resistance to check point inhibitors (CPI) (39).

## 2 Antibody-based therapeutic strategies

### 2.1 Antibodies against BTNA ligand

**Figure 2:**
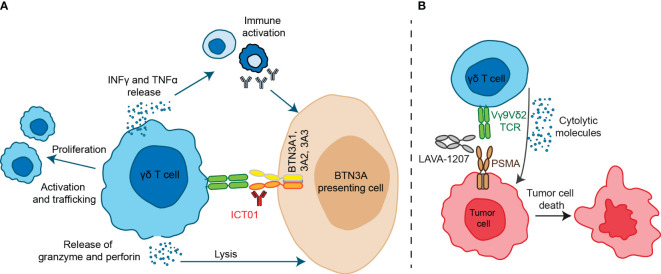
Antibody-based therapeutic strategies targeting Vγ9Vδ2 T cells. **(A)** ICT01 is a humanized monoclonal antibody which activates Vγ9Vδ2 T cells to kill tumor cells but not healthy cells. ICT01 has affinity for the three isoforms of BTN3A (BTN3A1, BTN3A2 and BTN3A3), but only BTN3A1 induces an extracellular conformational change when detects an intracellular pAgs increase. This conformational change is recognized by Vγ9Vδ2 T cells and this interaction induces Vγ9Vδ2 T cell activation and peripheral Vγ9Vδ2 T cell migration into tumor tissues. Besides, activation of Vγ9Vδ2 T cells induce TNF-α and IFN-γ production, leading to immune activation, as well as granzyme and perforin release which are mediators of cytolytic mechanisms. **(B)** LAVA-1207 is a humanized bispecific antibody that binds prostate-specific membrane antigen (PSMA) and the Vδ2 chain of Vγ9Vδ2-T cells, leading to lysis of PSMA-positive tumor cells in prostate cancer patients.

Antibodies designed against the butyrophilin BTNA ligand have been developed and recently have reached early clinical development. ICT01 is an antibody specifically designed to bind the BTNA ligand and induce a conformational change that permits the ligand to be in an activated position (40). ICT01 binds all three BTN3A isoforms (BTN3A1, BTN3A2, and BTN3A3) with *subnanomolar* affinities and a high degree of specificity; and displays little to no Fc effector functions (40). ICT01 acts independently of the B30.2 domain but requires the presence of the ligand to induce the synapsis (40) (**Figure 2A**).

Whether NKG2D or other receptors/ligands are involved in ICT01-induced selective cytotoxic activity toward cancer cells remains to be further elucidated. However, it has been suggested that activation of NKG2D is necessary for ICT01 to activate γδTCR through the BTN3A1 ligand. If there is no synapsis of NKG2D with its ligands, no activation of the γδTCR is produced and no effector function will appear (41).

In preclinical models ICT01 has demonstrated to efficiently activate Vγ9Vδ2T eliminating tumor cells *in vitro* and *in vivo* (40).

### 2.2 Clinical data

ICT01 has been evaluated in a first in human phase I study (EVICTION1 trial) with a monotherapy dose escalation part followed by a dose expansion monotherapy cohort in hematology tumors including, Acute Myeloid Leukemia (AML), Acute Lymphocytic Leukemia (ALL), Follicular Lymphoma (FL) and Diffuse Large B Cell Lymphoma (DLBCL); and an additional dose expansion cohort in combination with pembrolizumab in bladder cancer, Non-small Cell lung Cancer (NSCLC), Small Cell Lung Cancer (SCHNC) and melanoma (**Table 2**) (42). A total of 40 patients were treated in six different dose levels, with a maximum reached dose of 200 mg, and a recommended phase 2 dose of 75 mg. No dose limiting toxicities were identified and the safety profile was similar in all cohorts including the one in combination with pembrolizumab. Signs of T cell activation including cytokine release syndrome (CRS) and chills were observed at the 75 mg dose level. Clinical efficacy was detected with a clinical benefit rate (CBR) that ranged from 22% to 42% in combination with pembrolizumab in PD(L)1 pretreated patients (**Table 2**). An interesting observation was the association between the presence of Vγ9Vδ2T in peripheral blood and the infiltration of immune cells in the tumor, suggesting the potential association between peripheral blood counts and clinical activity (42) Of note, a potential limitation for targeting this receptor is the fact that they are also expressed on T cells, B cells, NK cells, and myeloid cells (26).

**Table 2:** Summary of clinical trials ongoing of antibody-based therapeutic strategies.

Study	Drug name	Protein type	Target	Phase	Route of administration	Cohort Indications	Dose level	Recomended dose
EVICTION-1	ICT01 +/- Pembrolizumab	Humanized anti- Butyrophilin 3A (BTN3A) monoclonal antibody	BTN3A	Phase 1	ICT01: Intravenous Pembrolizumab: intravenous	Acute Myeloid Leukemia (AML), Acute Lymphocytic Leukemia (ALL), Follicular Lymphoma (FL) and Diffuse Large B Cell Lymphoma (DLBCL) + Pembrolizumab: bladder cancer, Non-small Cell lung Cancer (NSCLC), Small Cell Lung Cancer (SCHNC) and melanoma patients who failed ≥1 checkpoint inhibitor (CPI)	ICT01: 20 μg to 200 mg Pembrolizumab: 200 mg	75 mg
EVICTION-2	ICT01 +/- low doses of IL-2	Humanized anti- Butyrophilin 3A (BTN3A) monoclonal antibody	BTN3A	Phase 1/2	ICT01: Intravenous IL-2: subcutaneous	Relapsed/refractory patients suffering from colorectal, ovarian, pancreatic or prostate cancer who had failed at least two lines of systemic therapy or had failed first line therapy	ICT01: 1 mg to 75 mg IL-2: 1 or 2 MIU/m2	ICT01 at 75 mg with IL-2 at 1 MIU/m2
LAVA051	LAVA-051 +/- low doses of IL-2	Vγ9Vδ2-T cell engaging bispecific antibody	CD1d and δ2- TCR chain of Vγ9Vδ2-T cells	Phase 1/2a	LAVA-051: Intravenous IL-2: subcutaneous	Chronic Lymphocytic Leukemia (CLL), Multiple Myeloma (MM), or Acute Myeloid Leukemia (AML)	0.45 μg to 100 μg	-
LAVA1207	LAVA-1207 +/- low doses of IL-2 or Pembrolizumab	Humanized bispecific antibody	Vd2 chain of Vγ9Vδ2-T cells and and to prostate specific membrane antigen (PSMA)	Phase 1/2a	LAVA-1207: Intravenous IL-2: subcutaneous Pembrolizumab: intravenous	Refractory metastatic castration resistant prostate cancer	1.5 μg to 120 μg	-

“-” means no reported.

### 2.3 Vγ9Vδ2T engagers

CD3 T cell engagers have demonstrated clinical activity in several indications (43, 44). However, this activity is also associated with a high presence of toxicity including CRS and the immune effector cell-associated neurotoxicity syndrome that clearly limits the clinical development (45, 46). Although different strategies have been implemented to improve the tolerability in patients, the safety profile of CD3 T cell engagers is still an important restriction (47, 48). Innate cells with inherent antitumoral activity like Vγ9Vδ2T could be used to create T cell engagers without the toxicity observed with CD3 T cell engagers, including CRS and off-tumor toxicity (49, 50).

Two Vγ9Vδ2T engagers have reached early clinical stage. LAVA051 is a Vγ9Vδ2T engager formed by fusing the CD1d-specific VHH1D12 with the Vd2-TCR-specific VHH5C8 (51). It binds the TCR of the Vγ9Vδ2T cells with CD1d that is a TAA expressed specifically in some hematological cells (52). In preclinical studies LAVA051 showed activation of Vγ9Vδ2T and an efficient *in vitro* and *in vivo* activity with an adequate safety profile (51). LAVA-1207 is a Fc-containing humanized bispecific antibody (~80 kD) that directly engages prostate-specific membrane antigen (PSMA) and the Vδ2-T cell receptor chain of Vγ9Vδ2-T cells (**Figure 2B**) (53). This Vγ9Vδ2T engager mediate potent killing of PSMA-expressing tumor cells (53).

A third Vγ9Vδ2T cell engager has been described but no clinical data has been reported yet. The EGFR- Vδ2 bsTCE is formed by single domain bi-specific antibodies (VHH, variable fragment of a heavy chain) that engage EGFR with Vδ2 TCR. Preclinical data suggested *in vitro* and *in vivo* activity (54). Of note, although EGFR is widely expressed in non-transformed tissue and previous EGFR-TCE showed toxicity, in this case the *in vivo* studies did not show toxicity in non-transformed tissue (54, 55).

In line with this, an interesting observation from preclinical studies was the fact that EGFR- Vδ2 bsTCE did not react against non-transformed cells expressing EGFR. This unexpected finding suggested that other mechanism of activation in the presence of tumoral cells was necessary to induce the effector function. Of note this could involve a second signal related to NKG2D, or a modulatory role of phosphoantigen/BTN3A, finding observed with a previous Vγ9Vδ2 TCE (56). In addition, upregulation of Tregs was not identified when evaluating Vγ9Vδ2 TCE, a differential finding compared with CD3 T cell engagers (57).

### 2.4 Clinical data

LAVA051 was evaluated in a phase 1/2a study in patients with chronic lymphocytic leukemia (CLL), multiple myeloma (MM), and acute myeloid leukemia (AML). It was administered intravenous or subcutaneously (s.c.) day 1 and 8 and thereafter twice a week (**Table 2**). The safety profile was adequate with no CRS or dose limiting toxicity (DLT), and most reported adverse event (AE) were not related to the study medication. LAVA051 pharmacokinetics (PK) was linear but very short, and signs of activation of Vγ9Vδ2T were detected through the evaluation of several biomarkers including CD25 and CD69. Of note in two patients early signs of clinical activity were detected (58). However, this clinical trial has been discontinued secondary to company strategic reasons mainly related to the competitive landscape in those indications, and not due to safety concerns (59).

LAVA1207 is in early clinical development in a phase 1/2a study in refractory metastatic castration resistant prostate cancer patients. Immunologic related reactions (IRRs) and CRS were observed at DL4 (>grade 2) so prophylactic administration of antipyretic or antihistamine was implemented (**Table 2**) (53). The pharmacokinetic (PK) profile appeared to be linear. Regarding signs of biological or clinical activity, LAVA1207 showed an elevation of Vγ9Vδ2T with expression of CD25 and CD69, and in two patients a reduction of PSA was observed (53).

### 2.5 Other Vγ9Vδ2T engagers in preclinical development

Other bi-specific formats have been developed but have not yet reached the clinical setting. For instance, it has been reported a bispecific γδ T cell engager (GADLEN), containing heterodimeric BTN2A1 and BTN3A1 extracellular domains (ECD) fused via inert Fc linkers to scFv domain targeting a tumor-antigen (CD19 or CD20) (60). Preclinical results have shown GADLEN activates Vγ9Vδ2+T cells and induces cell killing in the presence of NKG2D or CD28 co-stimulation (60). With this new format, again the presence of NKGD2 seems to be required for an adequate T cell activation. To our knowledge, these formats have not entered yet the clinical setting.

### 2.6 Options to improve the clinical development of antibody-based therapies targeting γδ T cells

There are two potential strategies that could improve the clinical development of antibody-based therapies targeting γδ T cells. They can be summarized in strategies to increase the expression of γδ T cells, or those aiming to boost the synapsis of γδ T cells with tumoral cells.

### 2.7 Increase the expression of γδ T cells

The observations from the EVICTION1 study suggested that ICT01 was able to induce an effector γδ T cell function in patients, and this effect correlated to the amount of Vγ9Vδ2T in peripheral blood (42). To overcome this problem, two strategies have been implemented. The first one, aimed to enrich the patient population of treated patients with those with the highest presence of Vγ9Vδ2T in peripheral blood. This approach was reported as a strategy to be implemented in the expansion cohorts of the ongoing EVICTION trial, where only patients with more than 5.000 cells per mm^3^ will be treated with the antibody (42). However, this approach has some limitations: first, there is a wide interpatient variability, so it is unclear the percentage of patients above this level; and secondly, it is unknown the cut-off level that will be associated with clinical activity. Identification of the correct level would require a large number of treated patients in different indications given the variability by tumor type and clinical scenario. Dose optimization strategies following FDA guidelines would be required to identify the right dose for the right population (61). To our knowledge, this strategy has been suggested but the identified findings have not been reported yet.

A further step is the administration of a therapeutic agent that will promote the expansion of Vγ9Vδ2T (**Table 2**). In the EVICTION-2 study the anti- BTNA ICT01 antibody was administered in combination with low dose SC IL-2. A dose escalation of ICT01 from 1 mg to 75 mg and two dose levels of sc IL2, 1 or 2MUI/m2 daily, on days 1-5 of cycles 1-3 were evaluated (62). The recommended dose level included a dose of ICT01 at 75 mg with IL2 at 1MUI/m2 sc. In all the eleven treated patients, an expansion of Vγ9Vδ2T was observed showing an activated profile with the presence of CD25, HLA-DR and PD1 (62). Following these findings, the EVICTION study has included an expansion cohort with the combination of ICT01, with low dose of IL-2 and pembrolizumab (63).

The lack of presence of Vγ9Vδ2T in peripheral blood can also be a limitation for the activity of Vγ9Vδ2 TCEs. To this regard, an expansion cohort in combination with low dose sc IL2 is planned for LAVA1207 in refractory metastatic castration resistant prostate cancer patients (64). In addition, an additional cohort in combination with pembrolizumab will be opened (63).

This strategy would not require a large number of treated patients, and given the fact that low dose IL2 sc can be considered as a non-therapeutic treatment, demonstration of the activity of each agent would not be required by regulatory authorities, and the combo treatment would be considered as a new therapy, in the same way as anti-CTLA4 priming strategies are combined with anti-PD (L)1 (65).

A main concern with the stimulation of IL2 is the presence of PD1 in Vγ9Vδ2T cells, a sign of potential exhausted T cells. This finding was observed in the EVICTION-2 study (62). This is the reason why anti-PD1 therapies like pembrolizumab are given in combination to anti-BTN antibodies or Vγ9Vδ2TCE plus IL2, in the ongoing studies. Of note, other interleukins (ILs) that could not induce an exhausted T cell phenotype can be explored like the administration of IL-15. At this moment several companies are developing superagonist fusion protein of interleukin (IL)-15 (66).

Beyond the administration of co-stimulatory ILs it has been suggested that the direct administration of allogenic γδ T cells could be an option. Although it is also known that ex-vivo expanded T cells can acquire an exhausted phenotype, recent studies with tumor infiltrating lymphocytes (TILs) have demonstrated clinical activity (67).

Finally, since BTN2A1 and BTN3A1 are not only expressed on tumor cells but also on Vg9Vd2 T cells and other T cells, the potential for (self-)elimination of immune cells should be taken into consideration when evaluating the safety profile and the presence of immune populations in peripheral blood (28).

### 2.8 Boosting the synapsis of γδ T cells with tumoral cells

Beyond the expansion of Vγ9Vδ2T cells other approaches could be explored. Given the fact that activation of NKG2D is key for a full activation of Vγ9Vδ2T cells, the use of activating antibodies against NKG2D in combination with either anti-BTN antibodies or γδ TCE could be evaluated (36, 68). In similar manner acting on MICA/B ligands could be used for therapeutic purposes (69). However, before implementing any of these strategies, it should be explored in patients already treated with antibodies against Vγ9Vδ2T cells if patients that did not respond lacked the expression of these receptors and ligands in the tumoral cells. This exercise could be easily done by the sponsored pharmaceutical companies.

## 3 Discussion

In the present review article, we describe the current knowledge of antibody-based therapies acting on Vg9Vd2 T cells. This therapeutic modality can undoubtedly overcome some of the limitations of classical immunotherapies like CPI or CD3TCE. In addition, the possible requirement of a second signal to activate T cells would offer the potential for better tolerability in non-transformed tissue where NKG2D ligands are not expressed. Unfortunately, the low number of Vγ9Vδ2T cells in peripheral blood seems to be a limitation to reach clinical activity in a higher number of patients. Ongoing strategies will demonstrate soon the utility of these new approaches like the co-administration of IL-2. Other options like the use of IL-15 could be explored.

## Author contributions

LP: Writing – original draft, Writing – review & editing. CD: Writing – original draft, Writing – review & editing. AS: Writing – original draft, Writing – review & editing. JB: Writing – original draft, Writing – review & editing. CN: Writing – original draft, Writing – review & editing. AO: Writing – original draft, Writing – review & editing.

## Glossary

**Table:**

AE	Adverse event
ALL	Acute lymphocytic leukemia
AML	Acute myeloid leukemia
BTNA	Butyrophilin
CBR	Clinical benefit rate
CLL	Chronic lymphocytic leukemia
CPI	Checkpoint inhibitors
CRS	Cytokine released syndrome
DLBCL	Diffuse large B cell lymphoma
DLT	Dose limiting toxicity
ECD	Extracellular domains
FL	Follicular lymphoma
IgV	Immunoglobulin V
IL	Interleukin
IRRs	Immunologic related reactions
MHC	Major histocompatibility complex
MM	Multiple myeloma
NK	Natural killer
NKT	Natural killer T
NSCLC	Non-small cell lung cancer
PAg	Phosphoantigens
PK	pharmacokinetics
PSMA	Prostate-specific membrane antigen
Sc	subcutaneously
SCHNC	Small Cell Lung Cancer
TAA	Tumor associated antigen
TCE	T cell engager
TCR	T-cell receptor
VHH	Variable fragment of a heavy chain.
